# Supplementary material for: Phototaxis Characteristics of Lymantria xylina (Lepidoptera: Erebidae)
Source: Insects. 2025 Mar 24;16(4):338. doi: 10.3390/insects16040338 (PMC12028050; doi:10.3390/insects16040338)
Supplement: Supplementary file 1 [file insects-16-00338-s001.zip › insects-3499888-supplementary.pdf]

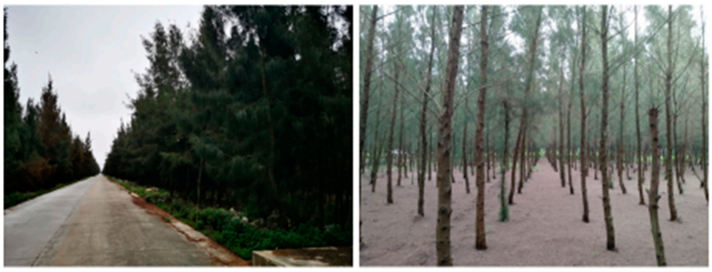

(a) (b)

Figure S1. The environment of the outdoor experimental site: **(a)** the insecticidal lamp hanging site and **(b)** the *Casuarina* forest.

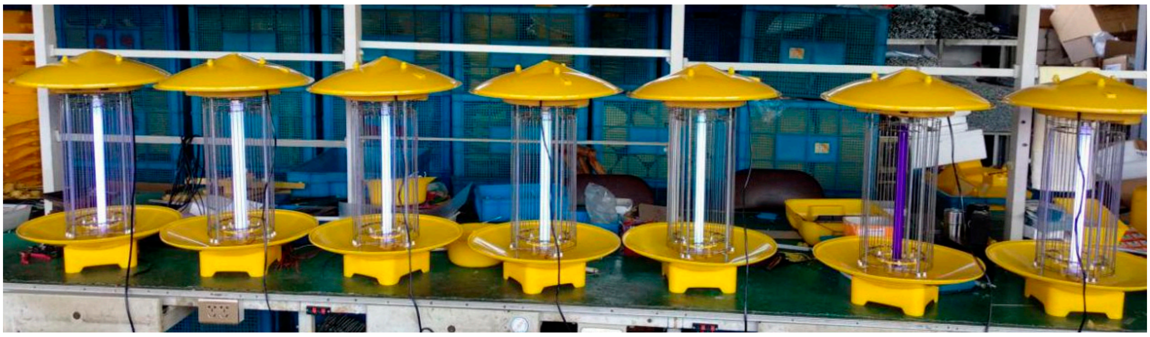

Figure S2. Part of the insecticidal lamps were assembled and tested in LOIHOI.

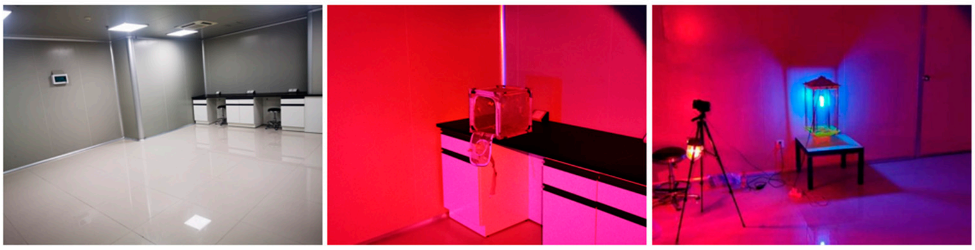

(a) (b) (c)

Figure S3. The phototactic behavior response tests of *L. xylina* adults in the darkroom: **(a)** the darkroom, **(b)** the net cages containing *L. xylina* adults, and **(c)** the L360 insecticidal lamp (25 W).

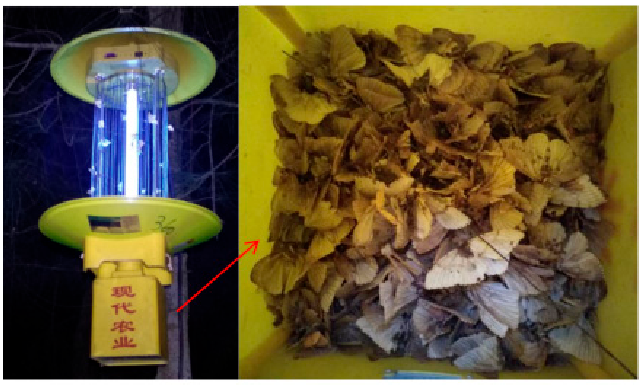

(a) (b)

Figure S4. The trapping effect of L360 insecticidal lamp on *L. xylina* adults: **(a)** moths stuck in a circular high-voltage grid and **(b)** moths fall into a collection box.

Table S1. The number of female and male *L. xylina* trapped by insecticide lamps at the peak period of emergence in 2019.

**No.**	**Central Wavelength (nm)**	**Type**	**Power (W)**	**Daily Trapping**										**Total Trapping**	
				**5/31 ***		**6/1 ****		**6/4**		**6/5**		**6/6**			
				**Female**	**Male**	**Female**	**Male**	**Female**	**Male**	**Female**	**Male**	**Female**	**Male**	**Female**	**Male**
L320	434	Fluorescent	15	0	89	1	8	0	185	0	144	0	39	1	465
L340	363	Fluorescent	15	0	508	1	12	0	206	0	127	0	64	1	917
L350	368	Fluorescent	15	0	597	1	10	1	201	2	238	0	142	4	1188
L360	363	Fluorescent	15	0	217	—	—	1	773	1	186	0	87	2	1263
L365	365	Fluorescent	15	0	571	1	13	0	234	1	212	0	56	2	1086
L370	368	Fluorescent	15	0	222	0	12	2	260	1	312	0	83	3	889
L380	378	Fluorescent	15	0	179	0	14	0	200	1	112	0	52	1	557
L405	439	Fluorescent	15	0	90	0	3	0	147	2	124	0	64	2	428
L_{LED}	365	LED	8	0	238	0	0	1	200	0	133	0	46	1	617
$L_{Full\ Spectrum}$	365	Full Spectrum	35	1	442	0	5	0	179	2	157	0	73	3	856
$L_{lighting\ lamp}$	550	LED	15	0	83	0	7	0	26	0	25	0	10	0	151
Total Trapping				1	3236	4	84	5	2611	10	1770	0	716	20	8417

Note: * represents cloudy to light rain; * * represents cloudy to moderate rain; grey represents the data used in the text. In addition, two female moths were trapped by the trapping effects of L360 insecticidal lamps with different powers (included in the text); one female moth was trapped by the trapping effects of L360 insecticidal lamps at different distances from the sea (unpublished data); three female moths were trapped from trapping effects between LED and fluorescent insecticidal lamps (unpublished data).

Table S2. The number of male *L. xylina* trapped by insecticide lamps at the late peak period of emergence in 2018.

Lamp	Central Wavelength (nm)	Type	Power (W)	Daily Trapping						Total Trapping
				6/26	6/27	6/28	7/3	7/4	7/5	
L320	434	Fluorescent	15	5	1	0	0	1	0	7
L340	363	Fluorescent	15	2	2	1	0	0	0	5
L350	368	Fluorescent	15	0	1	0	0	0	0	1
L360	363	Fluorescent	15	4	4	0	4	1	3	16
L365	365	Fluorescent	15	3	4	1	1	0	1	10
L370	368	Fluorescent	15	1	1	1	1	0	3	7
L380	378	Fluorescent	15	1	2	0	1	0	0	4
L405	439	Fluorescent	15	0	0	1	2	3	1	7

Note: Grey represents the data used in the text.
